# The Long Non‐Coding RNA Obesity‐Related (Obr) Contributes To Lipid Metabolism Through Epigenetic Regulation

**DOI:** 10.1002/advs.202401939

**Published:** 2024-05-05

**Authors:** Suneesh Kaimala, Shareena Saeed Lootah, Neha Mehra, Challagandla Anil Kumar, Saeeda Al Marzooqi, Prabha Sampath, Suraiya Anjum Ansari, Bright Starling Emerald

**Affiliations:** ^1^ Department of Anatomy College of Medicine and Health Sciences UAE University Al Ain P.O. Box 15551 UAE; ^2^ Department of Pathology College of Medicine and Health Sciences United Arab Emirates University Al Ain Abu Dhabi P.O. Box 15551 UAE; ^3^ A*STAR Skin Research Laboratory Agency for Science Technology & Research (A*STAR) Singapore 138648 Singapore; ^4^ Program in Cancer and Stem Cell Biology Duke‐NUS Medical School 8 College Road Singapore 169857 Singapore; ^5^ Genome Institute of Singapore Agency for Science Technology & Research (A*STAR) Singapore 138672 Singapore; ^6^ Department of Biochemistry and Molecular Biology College of Medicine and Health Sciences United Arab Emirates University Al Ain Abu Dhabi P.O. Box 15551 UAE; ^7^ Zayed Center for Health Sciences United Arab Emirates University Al Ain Abu Dhabi P.O. Box 15551 UAE; ^8^ ASPIRE Precision Medicine Research Institute Abu Dhabi Al Ain Abu Dhabi P.O. Box 15551 UAE

**Keywords:** cAMP response element binding protein, Creb histone acetyltransferase complex, diet‐induced obesity, lipid metabolism, lncRNA, obesity‐related

## Abstract

Obesity is a multifactorial disease that is part of today's epidemic and also increases the risk of other metabolic diseases. Long noncoding RNAs (lncRNAs) provide one tier of regulatory mechanisms to maintain metabolic homeostasis. Although lncRNAs are a significant constituent of the mammalian genome, studies aimed at their metabolic significance, including obesity, are only beginning to be addressed. Here, a developmentally regulated lncRNA, termed as obesity related (Obr), whose expression in metabolically relevant tissues such as skeletal muscle, liver, and pancreas is altered in diet‐induced obesity, is identified. The Clone 9 cell line and high‐fat diet‐induced obese Wistar rats are used as a model system to verify the function of Obr. By using stable expression and antisense oligonucleotide‐mediated downregulation of the expression of Obr followed by different molecular biology experiments, its role in lipid metabolism is verified. It is shown that Obr associates with the cAMP response element‐binding protein (Creb) and activates different transcription factors involved in lipid metabolism. Its association with the Creb histone acetyltransferase complex, which includes the cAMP response element‐binding protein (CBP) and p300, positively regulates the transcription of genes involved in lipid metabolism. In addition, Obr is regulated by Pparγ in response to lipid accumulation.

## Graphical abstract

The long non‐coding RNA obesity‐related (Obr) promotes H3K27 acetylation and transcriptional upregulation of genes involved in lipid metabolism through its association with the Creb histone acetyltransferase complex, which includes the cAMP response element‐binding protein (Creb), CREB‐binding protein (Cbp), and p300. Obr is regulated by Peroxisome proliferator‐activated receptor gamma (Pparγ) in response to lipid levels from the HFD or other sources.

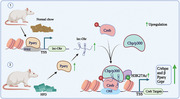

## 1 Introduction

A delicate balance between anabolic processes such as adipogenesis and lipogenesis and catabolic processes such as lipolysis, fatty acid β‐oxidation, and thermogenesis is needed to maintain energy balance.^[^ ^1^ ^]^ Obesity results from the imbalance in the calories of food ingested, and that which is expended.^[^ ^2^ ^]^ According to the World Health Organization, obesity is defined as the accumulation of excess fat in the body that interferes with the metabolism to the extent that it compromises health.^[^ ^3^, ^4^ ^]^ Obesity is also part of the metabolic syndrome, which includes other complications such as type 2 diabetes, cardiovascular diseases, and hypertension. It is increasing in epidemic proportion globally and has been associated with one in five deaths worldwide.^[^ ^3^, ^5^ ^]^

Metabolism is a highly regulated process that involves genetic, environmental, and other regulatory mechanisms and molecules, such as regulatory RNAs. Long noncoding RNAs (lncRNAs) are a class of regulatory RNAs with more than 200 nucleotides in length and are involved in various biological processes.^[^ ^6^ ^]^ In addition, lncRNAs may act directly to regulate the expression of nearby genes or serve as regulators directing the activity or localization of other proteins, and thereby, serve as organizational frameworks of subcellular structures; thus, functioning via numerous paradigms.^[^ ^7^ ^]^

As expected of regulatory molecules, lncRNAs are dysregulated in different diseases. These include cardiovascular diseases, neurological disorders, immune‐mediated diseases, genetic disorders, and various cancers.^[^ ^8^ ^]^ X‐inactive specific transcript (Xist) lncRNA, one of the first lncRNAs discovered, was shown to play an essential role in X chromosome inactivation.^[^ ^9^ ^]^ Further, a small number of lncRNAs has also been described that play a role in the control of adipogenesis (steroid receptor RNA activator (SRA), adipogenic differentiation induced noncoding RNA (ADINR), nuclear enriched abundant transcript 1 (NEAT1), MSTRG4710, and regulated in AdiPogenesis (lncRAPs) and the pathology of metabolic diseases, including DM2 (HI‐LNC25, KCNQ1 overlapping transcript 1 (KCNQ1OT1), LOC283177) and diabetic retinopathy (metastasis associated lung adenocarcinoma transcript 1 (MALAT1), Gm19619, high expression in M2 ATMs (HEM2ATM), growth arrest specific 5 (GAS5), and myocardial infarction associated transcript (MIAT)), have also been described.^[^ ^10^ ^]^ However, these are only a few examples as a single study on the analysis of nonalcoholic fatty liver disease transcriptome (NAFLD) alone has identified 535 lncRNAs that are upregulated and 1200 downregulated; although, the functional significance of these is not known.^[^ ^11^ ^]^ Therefore, although lncRNAs could play various regulatory roles in different metabolic processes and their changes lead to different diseases, the molecular regulatory mechanisms mediated by most of these lncRNAs are far from being understood.

The cAMP‐response element binding protein (CREB), a member of the leucine zipper superfamily of transcription factors, has been shown to mediate the effects of catecholamines and other fasting hormones on cellular gene expression.^[^ ^12^, ^13^ ^]^ Upon activation through its phosphorylation at Ser133, CREB binds to the cis‐acting CRE element within the promoters of the target genes together with histone acetyltransferases, cAMP response element‐binding protein‐binding protein (CBP), and p300 and is shown to activate their transcription.^[^ ^14^, ^15^ ^]^ Further, CREB has been shown to play a vital role in regulating glucose homeostasis as its expression is upregulated in diabetes, resulting in hyperglycemia and insulin resistance.^[^ ^16^ ^]^ It has also been shown that CREB plays a central role in the adipocytes' differentiation program, and their expression changes have been linked to obesity.^[^ ^17^ ^]^

Hepatic lipogenesis is regulated by well‐established transcription factors, which include Peroxisome proliferator‐activated receptor gamma (PPARγ) and CCAAT/enhancer‐binding protein family (C/EBP α, β), and changes in their expression have been linked to metabolic diseases, including obesity.^[^ ^18^ ^]^ Gastrin‐releasing peptide receptor (GRPR), which has been shown to regulate food intake in response to leptin signaling, has been identified as a gene whose expression is also altered in familial obesity.^[^ ^19^ ^]^ It has also been shown to be a target of CREB.^[^ ^20^ ^]^

From ongoing studies in our lab to identify novel lncRNAs with metabolic relevance, we have identified a developmentally regulated lncRNA, which we have termed obesity‐related (Obr) based on its regulatory role in lipid metabolism. We found that it is expressed in the tissues of metabolic relevance, such as the skeletal muscle, liver, and pancreas, and its expression is altered in diet‐induced obesity. Obr associates with the Creb and activates the critical transcription factors involved in lipid metabolism, such as Pparγ, C/ebp α, C/ebp β, as well the G protein‐coupled receptor, Grpr, through its association with CREB histone acetyltransferase complex. Obr is a target of Pparγ, which is active by HFD lipids, resulting in altered lipid metabolism and diet‐induced obesity.

## 2 Results

### 2.1 Expression of Obr is Upregulated in Diet‐Induced Obesity

**Figure 1:**
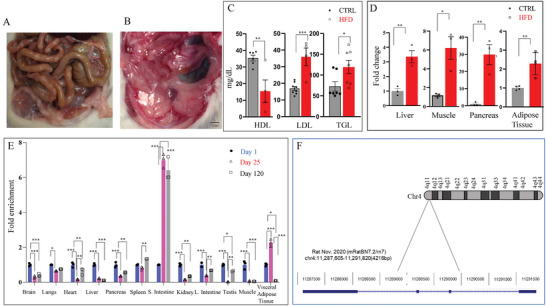
Expression of lncRNA *Obr* during the developmental stages and diet‐induced obesity in the liver, skeletal muscle (soleus and gastrocnemius), pancreas, and adipose tissue. A) The dissected abdominal area of control and B) HFD‐fed adult animals (day 120) depicting the internal organs covered with fat in the HFD‐fed animals. C) Serum HDL, LDL, and TGL levels of control and HFD‐fed day 120 adult animals. While the HDL levels were low, both LDL and TGL levels were significantly increased in the diet‐induced obese animals. D) qRT‐PCR analysis of the liver, skeletal muscle, pancreas, and adipose tissue showing the expression of Obr. The expression was upregulated in these tissues upon HFD‐induced obesity. E) Obr expression was high at birth in most tissues tested except the spleen, small intestine, and adipose tissue and was downregulated during the later developmental stages, days 25 and 120, in most of the tissues. In the small intestine and adipose tissue, the expression increased at day 25 and was downregulated at day 120. In the spleen, it decreased at day 25 and increased at day 120. F) In rats, Obr was located in chromosome 4q11. The lower panel shows the structure of the UCSC Genome Browser scheme (Rat Nov 2020, mRatBN7.2/m7). All qRT‐PCR experiments were performed in three independent experiments (*n* = 3), each in triplicate. Data were mean ± SE. *p < 0.05, **p < 0.01, and ***p < 0.001. Scale bar = 1 cm.

To understand the regulatory mechanisms altered in metabolic diseases and to identify the regulatory molecules including the lncRNAs, we are screening different published data sets relevant to metabolic diseases such as type 2 diabetes (T2DM) and obesity. From one of those earlier published transcriptomic data sets, we identified an uncharacterized lncRNA with multiple half cAMP response element (CRE) like sequences that we termed as obesity‐related (Obr) (GSE11492, Data S1, Supporting Information).^[^ ^21^ ^]^ In diet‐induced obese Wistar rats (Research diets, D12492) along with an increased fat accumulation (**Figure** **1**A,B) as known, the serum high‐density lipoprotein (HDL) level was low while both low‐density lipoprotein (LDL) and triglycerides (TCG) had increased (Figure 1C). The expression of Obr was significantly increased in the metabolically relevant tissues such as skeletal muscle (Soleus and Gastrocnemius), liver, pancreas, and adipose tissue from diet‐induced obese Wistar rats as assessed by quantitative real‐time PCR, which raised the possibility that Obr may have a role in lipid metabolism (Figure 1D).

### 2.2 The Expression of Obr in Tissues of Metabolic Relevance Changes During Development

Having seen an increase in the expression of Obr in metabolically relevant tissues in diet‐induced obesity, we next verified whether there were any changes in the expression of Obr during development using different tissues. We assessed its expression by qRT‐PCR at three developmental stages (day 1, day 25‐weaning, and day 120‐adult). We have seen that the expression of Obr was high in most of these tissues at 1‐day‐old pups, and its expression decreased during the later stages of development, days 25 and 120 (Figure 1E), suggesting the expression of Obr was developmentally regulated. The exceptions to this were the spleen, small intestine, and adipose tissues. In the small intestine and adipose tissues, the expression increased until day 25, and then, it decreased in the adults at day 120. In the spleen, increased expression was seen in the adults (day 120) (Figure 1E).

### 2.3 Obr is a Previously Uncharacterized lncRNA

As Obr is developmentally regulated and its expression is altered in diet‐induced obesity, we have used standard in slico methods to map the location of Obr toward understanding its functional significance. Obr mapped to the uncharacterized, predicted gene NR_110709.1 (Loc100909675) on chromosome 4q11. Based on the Rat, mRatBN7.2/rn7 assembly, Obr is predicted to produce a mature RNA of ≈1387 bp with four exons (Figure 1F).

### 2.4 Rat Obesity PCR‐Array Analysis Identifies Some of the Gene Targets of Obr

**Figure 2:**
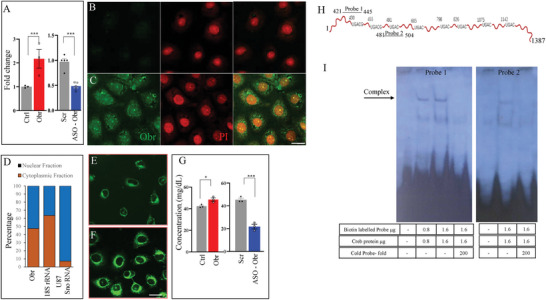
Obr is expressed in both the cytoplasm and the nucleus and alters lipid levels. A) qRT‐PCR showing the increased expression of Obr in C9 cells with stable overexpression and its down‐regulation with an ASO. C) In situ hybridization using an antisense Obr probe showing the localization to the cytoplasmic and nuclear compartments; B) while, there was no signal with a sense Obr probe. PI was used as a nuclear stain. D) Fractionation of nuclear and cytoplasmic RNA further confirms the presence of Obr in both the cytoplasmic and nuclear compartments. 18S RNA was used as a control for cytoplasmic fraction, and U87 Sno RNA was used as a nuclear marker. E,F) Increase in the level of lipid accumulation with increased expression of Obr in C9 cells with stable overexpression of Obr as seen with BODIPYTM 493/503 fluorophore (F) compared to control vector‐transfected cells (E). G) Quantitation of the neutral lipids showing the levels of neutral lipids increased with Obr overexpression and decreased with Obr downregulation. H) The RNA sequence of Obr shows the position of the eight half CRE‐like sequences and the location of the two RNA Probes 1 and 2 used for the RNA EMSA. I) RNA EMSA showing the Obr complex with Creb Protein. This complex competes with cold RNA probes (Probe 1, lane 4 and Probe 2, lane 3). Data are mean ± SE. *p < 0.05, **p < 0.01, and ***p < 0.001. Scale bar = 50 µm.

To gain insight into the genes that might be regulated by Obr and the possible regulatory mechanism that may be contributing to the changes observed in diet‐induced obese animals, we have used the Rat obesity RT2 Profiler PCR array. This array includes 84 genes that are known to be associated with energy balance, including neuropeptides and receptors, gut hormones and receptors, adipocyte‐derived peptides and receptors, pancreas‐derived peptides and receptors, and CNS‐derived peptides and receptors. To identify the targets of lncRNA‐Obr, we have cloned the Obr as described in the Experimental Section and generated a normal rat liver cell line (clone 9 [ATCC CRL‐1439]) with stable expression of Obr (**Figure** **2**A). Vector‐transfected control cell lines were also generated, as described in the Experimental Section (Figure 2A). We used the cDNAs from these cell lines and performed PCR array analysis to identify those targets that changed due to changes in the expression of Obr.

Our results revealed 23 genes which included, Attractin (Atrn), Brain‐derived neurotrophic factor (Bdnf), Complement component 3 (C3), Growth hormone receptor (Ghr), Gastrin releasing peptide receptor (Grpr), Hypocretin (orexin) receptor 1 (Hcrtr1), Interleukin 1 receptor type I (Il1r1), Interleukin 6 receptor (Il6r), Neuromedin B (Nmb), Neuromedin U (Nmu), Neuropeptide Y (Npy), Peroxisome proliferator‐activated receptor alpha (Pparα), Peroxisome proliferator‐activated receptor‐gamma (Pparγ), Sigma non‐opioid intracellular receptor 1 (Sigmar1), Sortilin 1 (Sort1), Thyrotropin‐releasing hormone (Trh), Ciliary neurotrophic factor receptor (Cntfr), Insulin 2 (Ins2), Leptin receptor (Lepr), Neuromedin U receptor 1 (Nmur1), Opioid receptor, kappa 1 (Oprk1), Proopiomelanocortin (Pomc), and Thyroid hormone receptor beta (Thrβ) that were significantly and differently expressed in Obr overexpression cells after normalizing using different housekeeping genes provided by the arrays (*p* < 0.05). Of these, 16 genes (Atrn, Bdnf, C3, Ghr, Grpr, Hcrtr1, Il1r1, Il6r, Nmb, Nmu, Npy, Pparα, Pparγ, Sigmar1, Sort1, and Trh) were upregulated and 7 genes (Cntfr, Ins2, Lepr, Nmur1, Oprk1, Pomc, and Thrβ) were down‐regulated in Obr overexpression cells (Figure S1A,B, Supporting Information).

The differentially expressed genes were grouped according to functional significance using the gene set enrichment analysis (GSEA). Genes enriched for each pathway were further grouped together. The ten different pathways to which these genes were grouped included the adipokine signaling pathway, regulation of lipolysis in adipocytes, nonalcoholic fatty liver diseases (NAFLD), insulin resistance, insulin signaling pathway, and T2DM (Figure S1C, Supporting Information).

### 2.5 Quantitative Real‐Time PCR Validates the Targets of Obr

We performed qRT‐PCR experiments using the same RNA samples from cell lines that overexpress Obr to validate the PCR‐array expression results. Five of the genes which were upregulated in the rat liver cell line (C9), namely Grpr, Ghr, Trh, C3, Pparγ, and one gene whose expression was down‐regulated, Nmur1, were found to be upregulated or downregulated by qRT‐PCR, complementing the results of the PCR‐array (Figure S1D, Supporting Information)

To further verify the results obtained through the PCR array, we have also generated antisense oligonucleotides (ASOs) for Obr, described in the Experimental Section, and transfected the rat liver cell line, clone 9 with the ASOs. Clone 9 cells with control ASO were also generated (Figure 2A). Of the two different ASOs generated (Experimental Section; Table S1, Supporting Information) and tested, Obr ASO2 was found to be the most effective with respect to the down‐regulation of Obr expression, and so we used it in all the experiments (Figure 2A). We extracted RNA from the control‐ASO and Obr ASO2 C9 cell lines and performed qRT‐PCR analysis to verify the target genes whose expression was altered in response to Obr down‐regulation. We verified five of those genes, Grpr, Ghr, Trh, C3, Pparγ, and Il6, whose expression was upregulated with increased expression of Obr and found them to be down‐regulated when the expression of Obr was down‐regulated (Figure S1E, Supporting Information). Therefore, the results complemented those obtained from the PCR array, suggesting that these genes were genuine targets of lncRNA Obr. We also generated the possible association of these genes with respect to metabolism, which is given in Figure S1F, Supporting Information.

### 2.6 Obr is Expressed in Both the Cytoplasm and Nucleus

Having seen changes in the expression of multiple genes with changes in the expression of Obr, we verified the localization of Obr expression by fluorescence in situ hybridization using antisense and sense Obr probes as described in the Experimental Section. Propidium iodide was used as the marker for the nucleus. Our results showed that Obr was expressed in the nucleus and cytoplasm (Figure 2C); while, we didn't see any staining with the sense control probe (Figure 2B). To verify these further, we generated nucleus and cytoplasmic RNA fractions and verified the expression of Obr in these fractions, which further showed the expression of Obr in both the cytoplasmic and nuclear compartments (Figure 2D). 18S RNA and U87 snoRNA were used as markers for the cytoplasmic and nuclear fractions (Figure 2D).

### 2.7 Obr Associates With Creb and Alters Lipid Levels

As different genes involved in lipid metabolism/obesity are changed as a result of the Obr expression, it raised the possibility that Obr may be a part of a higher‐order gene regulatory mechanism that targets key transcription factors involved in lipid metabolism. To verify this possibility, we tested first whether there is any visible increase in lipid accumulation with increased expression of Obr by using C9 cells with stable overexpression of Obr and compared with vector‐transfected control cells by staining with the fluorophore, 4,4‐Difluoro‐1,3,5,7,8‐Pentamethyl‐4‐Bora‐3a,4a‐Diaza‐s‐Indacene (BODIPYTM 493/503), which had been shown to stain the neutral lipids.^[^ ^22^ ^]^ We could see an apparent increase in the accumulation of lipids in those cells with overexpression of Obr (Figure 2E,F). We also quantitated the levels of neutral lipids by using C9 cells with stable overexpression of Obr or C9 cells, which were transfected with the Obr ASOs, and compared their expression to vector‐transfected control cells. The levels of neutral lipids increased with Obr overexpression and decreased with Obr downregulation (Figure 2G).

Earlier studies have shown that one of those transcription factors that can induce de novo lipogenesis and adipogenesis is CREB.^[^ ^23^ ^]^ Creb has been shown to bind via a conserved gene promoter element, cAMP response element (CRE) (TGACGTCA), or half‐sites (TGACG or CGTCA) to specific gene promoters and regulate their expression.^[^ ^24^, ^25^ ^]^ One of the factors that prompted our interest in the lncRNA Obr was the presence of eight half CRE‐like sequences, which raised the possibility that Obr may bind directly to Creb protein (Figure 2H). The secondary structure of Obr with the half CREs is given in Figure S2, Supporting Information. We generated RNA probes covering two possible CREs (421‐442 probe 1 and 481–504) and performed RNA‐EMSA using rat Creb protein to verify this. Our results showed the formation of the complex between Obr and Creb proteins, and this interaction could be competed out by adding a cold Obr probe, suggesting that Obr associates with the Creb (Figure 2I).

### 2.8 Obr Associates With the Creb Histone Acetyltransferase Complex

Earlier studies have shown that the activation of CREB is through its phosphorylation at Ser133 by activated PKA. CREB forms a transcriptional activation complex by associating with the histone acetyltransferases, CBP and p300, and RNA polymerase II, which binds the promoters of target genes that contain the CREs.^[^ ^24^ ^]^ Binding of CREB histone acetyltransferase complex increases expression of downstream target genes by opening up the chromatin and increasing their accessibility to RNA polymerase.^[^ ^26^ ^]^

**Figure 3:**
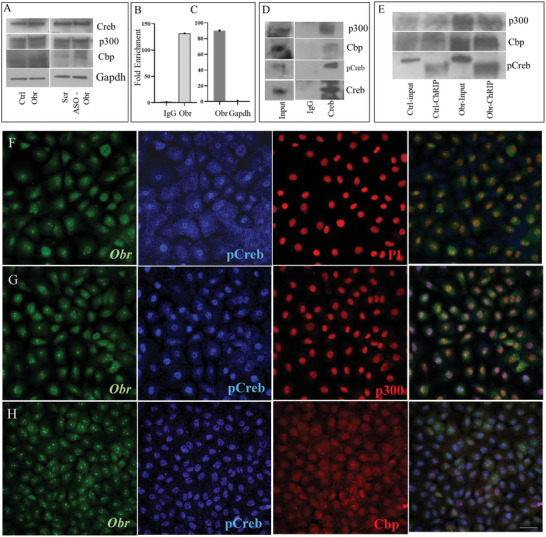
Obr associates with the Creb histone acetyltransferase complex. A) C9 cells stably overexpress, or the expression of Obr downregulated using ASOs showing the expression of Creb and histone acetyltransferases Cbp and p300. No change in the expression was observed by western blot analysis. Gapdh expression was used as the loading control. B) RNA immunoprecipitation (RIP) analysis shows that a significant amount of Obr is immunoprecipitated using an anti‐Creb antibody compared to IgG control, suggesting that Obr associates with Creb. C) Fold enrichment of Obr to that of Gapdh by RIP establishes the specificity of RIP. D) RIP analysis of C9 cells shows that Creb and histone acetyltransferases Cbp and p300 were co‐immunoprecipitated using an anti‐Creb antibody. IgG was used as control, and 10% was used as Input. E) Representative blot of ChRIP analysis of C9 cells with vector control or overexpression of Obr shows that Creb and histone acetyltransferases Cbp and p300 were co‐immunoprecipitated using an Obr antisense probe. Western analysis with pCreb, Cbp, and p300 antibodies shows all were co‐immunoprecipitated, and their level increased with over‐expression. 10% was used as input (lanes 1 and 3). The colocalization of Obr with the pCreb and histone acetyltransferases Cbp and p300 was analyzed using in situ hybridization of Obr followed by pCreb and PI as a nuclear marker F), Obr in situ, followed by pCreb and p300 immuno staining G) and H) Obr in situ followed by pCreb and Cbp immunostaining. Obr was colocalized with pCreb, p300, and Cbp in the nucleus. Scale bar = 50 µm.

So, we tested whether there was any change in the expression of Creb and the histone acetyltransferases, Cbp and p300, by using C9 cells with overexpression of Obr or knockdown using ASOs using western blotting. We did not see any significant difference in the expression of Creb, Cbp or p300, between Obr overexpression or knockdown and control C9 cells, suggesting it is not transcriptionally activating Creb, Cbp, and p300 (**Figure** **3**A).

We next performed an RNA immunoprecipitation (RIP) analysis to verify the possibility that Obr may be associating with Creb and interacting with the different targets of Creb and contributing to lipid accumulation. We used a specific anti‐Creb antibody to pull down the associated RNA and protein complexes and verified the association by qRT‐PCR or western blotting. We immunoprecipitated a significant amount of Obr by RNA immunoprecipitation using an anti‐Creb antibody, compared to IgG control, confirming that Obr interacts with Creb (Figure 3B). We further verified the fold enrichment of Obr to that of Gapdh to establish the specificity of our RNA immunoprecipitation. Compared to Gapdh, we have seen more than 90‐fold enrichment of Obr in the Creb RIP, further confirming that Obr interacts with Creb (Figure 3C).

As we have seen, the Creb antibody was able to immunoprecipitate Obr; we verified using C9 cells whether Obr is also part of the Creb histone acetyltransferase complex by RIP using pCreb antibody and tested the immunoprecipitate for the presence of pCreb, CBP, and p300 proteins by western blotting. IgG was used as the control. Our results show that the immunoprecipitate contained the histone acetyltransferases, CBP, and p300, along with pCreb and Obr (Figure 3D). To independently verify the existence of the Obr, Creb histone acetyltransferase protein complex, we have performed a Chromatin Isolation via RNA Precipitation (ChIRP) analysis, which is an antisense probe‐based capture strategy that uses crosslinked chromatin to allow unbiased discovery of RNA‐associated DNA sequences and proteins. We have generated multiple biotinylated antisense probes for Obr (Table S3, Supporting Information), combined them with chromatin from C9 control cells and those with stable overexpression of Obr, and hybridized them to the chromatin‐associated RNA. Complexes containing biotinylated probes bound to the chromatin‐associated regions of Obr were then isolated using streptavidin magnetic beads. Proteins were isolated and assessed by western blotting. 10% was used as input. Our results again confirmed the presence of histone acetyltransferases, CBP, and p300, along with pCreb, in the immunoprecipitate, and their level increased with overexpression of Obr (Figure 3E). These results indicated that Obr associated with the Creb histone acetyltransferase complex.

We also further verified the colocalization of the Obr with that of the Creb histone acetyltransferase protein complex, CBP, p300, and pCreb by in situ hybridization of Obr in C9 cells followed by immune staining. Our results showed the co‐localization of histone acetyltransferases, CBP, p300, and pCreb, with Obr in the nucleus (Figure 3F–H). PI staining was used as the marker for the nucleus (Figure 3F). The colocalization of Obr with histone acetyltransferases, CBP, and p300 is shown in Figure S3, Supporting Information.

### 2.9 Obr Promotes the Transcription of Genes Involved in Lipid Metabolism

As we found that Obr interacts with the active Creb histone acetyltransferase complex, this raised the possibility that Obr may regulate key transcription factors involved in lipid metabolism, which are Creb targets. Further, CREB is a nuclear protein that binds to the CRE sequences, and the CREs are located in the proximal promoter regions of genes in most cases.^[^ ^13^, ^27^ ^]^

**Figure 4:**
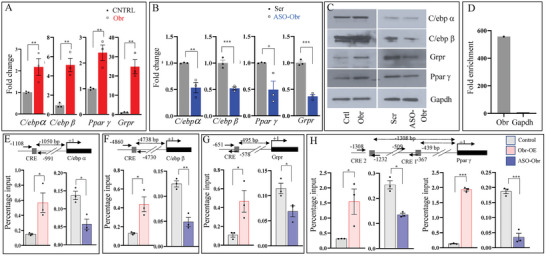
Obr promotes the expression of transcription factors involved in lipid metabolism. The expression analysis of C/ebpβ, Pparγ, C/ebpα (A) Grpr (2E) by qRT‐PCR of C9 cells, which stably overexpress Obr A) show an increase in their expression with increased expression of Obr and B) a decrease in their expression when the expression of Obr is down‐regulated by ASOs (Figure S1F, Supporting Information). Vector transfected cells were used as the control for overexpression, and Scrambled RNA (scr) was used as a control for ASOs. C) Western blot analysis of the same genes showing the change in their expression is similar to what was observed with qRT‐PCR with stable overexpression or down‐regulation of Obr expression. D) ChIRP analysis using the antisense probe‐based capture strategy showing fold enrichment of Obr to Gapdh. E–H) ChIRP analysis of the binding of Obr to the CRE sites of the genes C/ebpβ, Pparγ, C/ebpα, and Grpr. Changes in the expression of Obr using over‐expression or down‐regulation using ASO affects the binding of Obr (E–H). The region analyzed is shown above the histograms. Data are mean ± SE. *p < 0.05, **p < 0.01, and ***p < 0.001.

One critical transcription factor involved in lipid accumulation is C/EBPβ, and it has been shown to play a central role in lipid metabolism through its subsequent induction of transcription PPARγ and C/EBPα.^[^ ^28^ ^]^ Similarly, another gene that regulates lipid metabolism is GRPR, and the inhibition of its expression was shown to reduce the expression of C/EBPβ and PPARγ.^[^ ^29^ ^]^ We have verified the expression of these genes, C/ebpβ, Pparγ, C/ebpα, and Grpr, by both qRT‐PCR for RNA and Western blotting for protein expression levels using C9 cells with overexpression or downregulation of Obr using ASO. Our results showed that the expression of all these genes was upregulated or downregulated in response to changes in the expression of Obr (**Figure** **4**A–C).

We then verified the possibility that Obr interacts with these genes of lipid metabolism, C/ebpβ, Pparγ, C/ebpα, and Grpr, as part of the active Creb histone acetyltransferase complex. We performed ChIRP analysis and verified the RNA‐associated DNA CRE sequences. First, to establish the specificity of our ChIRP analysis, we verified the fold enrichment of Obr to that of Gapdh by normalizing it with the input. Compared to Gapdh, we have seen around 600‐fold enrichment of Obr in the Obr ChIRP, which established the specificity of our experiment (Figure 4D).

We then tested the binding of Obr to the CRE sites of these genes C/ebpβ, Pparγ, C/ebpα, and Grpr using the ChIRP recovered DNA by qRT‐PCR. Our results showed an apparent increase in the binding of Obr to the CRE sites of these genes when there was forced expression of Obr, and this binding was reduced when the expression of Obr was down‐regulated with ASOs (Figure 4E–H). These results suggest that Obr promotes the transcription of genes involved in lipid metabolism through its interaction with Creb, and possibly, by interacting with the CRE sites at the promoters of these genes.

### 2.10 Expression Changes in Obr Affect Histone Acetylation

As earlier experiments suggested that Obr may be part of the Creb histone acetyltransferase complex and changes in its expression affected the expression of genes, C/ebpβ, Pparγ, C/ebpα, and Grpr, we next examined whether this also changed the level of H3K27 acetylation, one of the histone tail modifications orchestrated by this complex. We have used the C9 liver cells with increased or decreased expression of Obr along with their respective vector controls and performed chromatin immunoprecipitation (ChIP) analysis using an antibody specific for H3K27 acetylation modification. We also evaluated the total acetylation levels in these cells by ChIP analysis.

**Figure 5:**
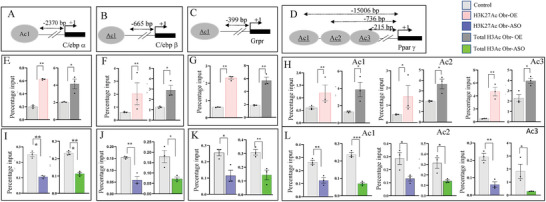
Changes in the expression of Obr affect histone acetylation. C9 liver cells with increased expression of Obr and those where the expression of Obr was down‐regulated by ASOs, along with the respective vector or scr controls, were used to perform chromatin immunoprecipitation (ChIP) analysis. A) For C/ebpα, we verified the promoter region around −2370 bp upstream of the start site, B) for C/ebpβ around −665 bp upstream of the transcription start site, C) for Grpr around −399 bp upstream of the transcription start site, and D) for Pparγ −215 bp, −736, and −1506 bp upstream of the transcription start sites. We assessed the total H3 acetylation and the levels of H3K27 acetylation using the cells with E–H) increased or I–L) downregulation of Obr expression in promoters of the genes tested: C/ebpα, C/ebpβ, Grpr, and Pparγ, using an antibody specific for H3K27 acetylation or total H3 using ChIP analysis. Both the total H3 and the levels of H3K27 acetylation showed an increase or decrease, which correlated with the expression change of Obr. Data are given as mean ± SE. *p < 0.05, **p < 0.01, and ***p < 0.001.

For C/ebpα we verified the promoter regions around −2370 bp upstream of the start site, for C/ebpβ, −665 bp upstream of the transcription start site, for Grpr, around −399 bp upstream and for Paprγ −215 bp, −736, and −1500 bps upstream of the transcription start site (**Figure** **5**A–D). For all these genes, H3K27 and the total H3 acetylation correlated positively to the increase in Obr expression (Figure 5E–H). We also assessed the H3K27 and total H3 acetylation using C9 cells where Obr expression was down regulated using ASOs, and both these modification levels were decreased when Obr expression was down regulated (Figure 5I–L). The results were shown as percentage input after normalizing to IgG. These results suggested that the expression changes seen in C9 cells with overexpression and downregulation of the expression of Obr may be a result of changes in the levels of H3K27 acetylation at promoters of these genes, and the Obr might be promoting transcriptionally active chromatin as a member of the Creb histone acetyltransferase complex.

### 2.11 Obr is a Member of the In Vivo Creb Histone Acetyltransferase Complex and is Altered in Diet‐Induced Obesity

**Figure 6:**
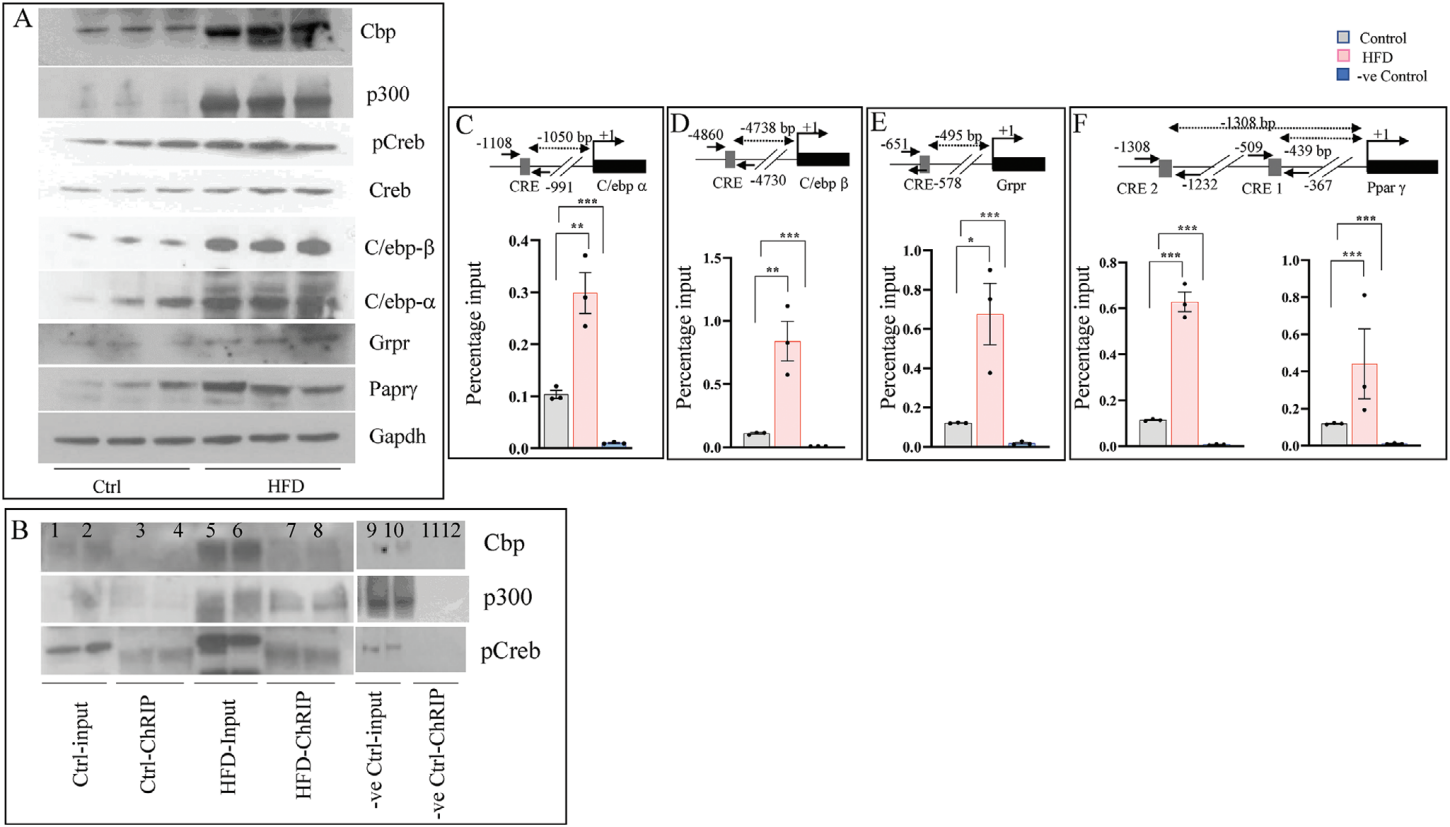
Obr is a member of the Creb histone acetyltransferase complex in vivo and is altered in diet‐induced obesity. Liver tissues from normal and HFD‐fed Wistar rats, as described in the Experimental Section, were used to assess the expression of the regulatory genes of lipid metabolism, Crebp, C/ebpβ, Pparγ, C/ebpα, Grpr, pCreb, and the acetyltransferases Cbp and P300 by western blotting. Gapdh was used as a loading control. A) The expression of these genes was increased in the livers of HFD‐fed Wistar rats compared to the chow‐fed control rats. B) ChRIP analysis using livers from the control or HFD‐fed Wistar rats shows that Creb and histone acetyltransferases Cbp and p300 were co‐immunoprecipitated using an anti‐Obr probe. Western blot analysis with pCreb, Cbp, and p300 showed all were precipitated, and their level increased in the levers of HFD‐fed animals. ChRIP analysis using a Gapdh probe was used as a negative control. 10% was used as input (lanes 1,2, 5,6, and 9,10). C–F) ChRIP analysis using the liver tissue from rats fed either with normal chow or with HFD showed increased binding to the promoters of C/ebpα,  C/ebpβ, Grpr, and Pparγ. The region verified is shown above. The results show increased binding to the promoters of all these genes. Gapdh primers were used as a negative control for the ChRIP analysis.

As mentioned, it has been shown that Creb interacts with the acetyltransferases Cbp and P300 and positively regulates the transcription, including those involved in lipid metabolism.^[^ ^23^, ^30^ ^]^ Therefore, to determine the functional relevance of Obr as a member of the CREB histone acetyltransferase complex in vivo, we used the liver from normal and HFD‐fed Wistar rats as described in the methods and determined the expression of the regulatory genes of lipid metabolism, Crebp, C/ebpβ, Pparγ, C/ebpα, and Grpr, along with pCreb and the acetyltransferases Cbp and P300 by western blotting. Gapdh was used as the loading control. Our results showed that the expression of all these genes was increased in the livers of HFD‐fed Wistar rats compared to those of chow‐fed rats (**Figure** **6**A).

To verify the existence of the in vivo Obr, Creb histone acetyltransferase protein complex, we have performed the ChIRP analysis using multiple biotinylated antisense probes for Obr using the liver tissue of rats fed either with normal chow or with HFD. Proteins were isolated and assessed by Western blotting. 10% was used as input. Our results again confirmed the presence of the in vivo Creb histone acetyltransferase complex as we could immunoprecipitate CBP and p300 along with pCreb. Their level also increased in the livers of HFD diet‐fed animals where there was overexpression of Obr (Figures 1E and 6B). As negative controls, ChRIP assays were performed using Gapdh probes (Figure 6B).

Using the liver tissues of Wistar rats fed with normal chow or HFD, we tested the binding of Obr to the CRE sites of these genes C/ebpβ (−4730), Pparγ (−367, −1232), C/ebpα (−991), and Grpr (−578) using the ChIRP recovered DNA by qRT‐PCR (Figure 6C–F). Our results showed an apparent increase in the binding of Obr to the CRE sites of these genes in the livers of HFD‐fed animals. qRT‐PCR of ChIRP recovered DNA using Gapdh primers, which were used as the negative control. These results further supported the possibility that Obr interacted with the CRE sites in the promoters of these genes and increased when there was an increased expression, as in the livers of HFD‐fed animals.

**Figure 7:**
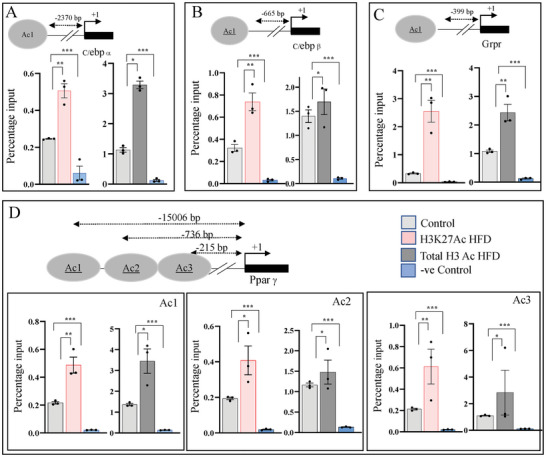
Histone acetylation is affected in the livers of HFD induced obese animals. A–D) ChIP analysis of the H3 total acetylation and the H3K27 acetylation levels using the livers of control and HFD‐fed animals. We have used the same promoter regions described for the C9 cells (≈ −2370 bp for C/ebpα, Pparγ −665bp for C/ebpβ, around −399 bp for Grpr and −215 bp, −736 and −1506 bps upstream for Pparγ).  The results showed an enrichment of H3K27 acetylation and total H3 acetylation. IgG ChIP was used as a negative control. Data are given as mean ± SE. *p < 0.05, **p < 0.01, and ***p < 0.001.

We then performed ChIP analysis using antibodies specific for H3K27 acetylation and H3 total acetylation to verify the changes in acetylation levels in vivo. We tested the binding of Obr to the same promoter regions (around −2370 bp for C/ebpα, −665bp for C/ebpβ, around −399 bp for Grpr and −215 bp, −736 and −1500 bps upstream for Paprγ). The results were shown as percentage input. IgG ChIP was used as the negative control. Our results show an apparent increase in both H3K27 and H3 total acetylation at the CRE sites of these genes C/ebpβ, Pparγ, C/ebpα, and Grpr in the livers of HFD‐fed animals (**Figure** **7**A–D). These results further supported the idea that Obr promotes the transcription of genes involved in lipid metabolism, possibly through its interaction with the CRE sites in the promoters as part of the Creb histone acetyltransferase complex in vivo as well.

### 2.12 Pparγ Activates Obr

**Figure 8:**
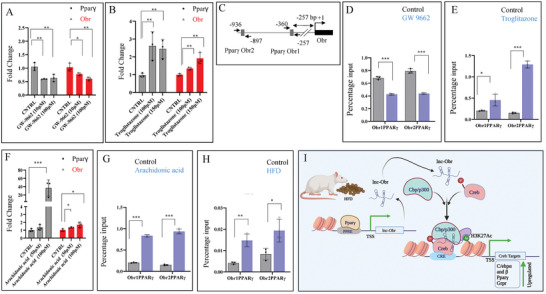
Obr is a target of Pparγ, and the HFD diet induces Obr expression through Pparγ. A,B) C9 cells were treated with two different concentrations of Pparγ antagonist (Sw‐9662) or agonist Troglitazone, and the expression of Pparγ and Obr was analyzed by qRT‐RCR. The expression of Obr decreased (A) or increased (B) in response to changes in Pparγ expression. C) The Pparγ response elements (PREs) regions were tested in the Obr promoter. D,E) C9 liver cells were treated with Pparγ antagonist (Sw‐9662) or the agonist Troglitazone, and the binding of Pparγ to two different PRE‐regions of the Obr promoter was analyzed by ChIP analysis. The binding decreased in the presence of the antagonist (D) and increased with the activation of Pparγ (E). F) C9 cells were treated with two different concentrations of polyunsaturated lipid, Arachidonic acid, which is present in HFD, and the expression of Pparγ and Obr was analyzed by qRT‐RCR. Both the expression of Obr and Pparγ were increased in response to the addition of Arachidonic acid. G) C9 cells were treated with polyunsaturated lipid, Arachidonic acid as above, and the binding of Pparγ to two different PREs mentioned above was tested by ChIP analysis. The binding increased in the presence of Arachidonic acid. H) Using liver tissue samples from normal chow and HFD‐fed animals, we have also verified the binding of Pparγ to the PREs of the Obr promoter mentioned above by ChIP analysis. The binding of Paprγ to the Obr promoter was increased in the livers of HFD‐fed animals. I) The model depicts the association of Obr with the CREB histone acetyltransferase complex. An increase in the lipid levels from the HFD or other sources increased the expression of Pparγ, which increased the expression of Obr. Obr associated with the CREB histone acetyltransferase complex, and this association promoted H3K27 acetylation at promoters of H3K27 acetylation at promoters of the target genes. Data are given as mean ± SE. *p < 0.05, **p < 0.01, and ***p < 0.001.

To understand the mechanism which regulates the expression of Obr, we have analyzed the promoter region of Obr. We have identified two possible binding sites, Paprγ response elements (PREs), for the known transcription factor involved in regulating lipid metabolism Pparγ at (−257 to – 360 and −897 to −936) of the Obr promoter (**Figure** **8**C). Earlier studies have shown that Pparγ mediates high‐fat diet‐induced lipid accumulation.^[^ ^31^ ^]^ We have verified the possibility that Pparγ may be regulating the expression of Obr by treating the C9 cells with the Pparγ agonist, Troglitazone and antagonist, SW‐9662, and verified the expression of both Pparγ and *Obr* by qRT‐PCR. The expression of Obr was increased in cells where the expression of Pparγ was increased by the agonist (Figure 8B). In contrast, its expression was downregulated, where the expression of Pparγ was downregulated by the antagonist (Figure 8A). We have also tested the binding of Pparγ to the Obr promoter PRE‐sites after treating the C9 cells with the Pparγ agonist, Troglitazone or antagonist, SW‐9662, and verified the binding by ChIP analysis. Our results show an apparent increase in the binding of Pparγ to the Obr CRE sites with an increase in the expression of Pparγ, and the binding decreased with the downregulation of the expression of Pparγ, suggesting that Pparγ directly bound the promoter of the Obr and activated its transcription (Figure 8D,E).

As we saw the level of Obr was increased with diet‐induced obesity, and the transcription factor Pparγ involved in lipid metabolism regulated the expression of Obr, we verified the possible mechanism by which Obr was upregulated in response to HFD. It has been shown that fatty acids in the HFD activate Pparγ.^[^ ^32^, ^33^ ^]^ So, we verified this possibility by treating the C9 cells with arachidonic acid, a polyunsaturated fatty acid possessing 20 carbon atoms and four double bonds (C20: 4) and verified the expression of both Pparγ and Obr. Increased levels of the polyunsaturated fatty acid Arachidonic acid increased the expression of both Pparγ and Obr (Figure 8F), suggesting one possibility of Obr expression increase in diet‐induced obesity. We also tested whether increased levels of the polyunsaturated fatty acid Arachidonic acid also increased the binding of Paprγ to the Obr promoter by ChIP analysis. As seen with the expression, increased levels of the polyunsaturated fatty acid Arachidonic acid increased the binding of Paprγ (Figure 8G).

We have also tested the binding of Pparγ to the Obr promoter PRE‐sites (−257 to – 360 and −897 to −936) using ChIP analysis of control and HFD‐induced obese animal liver tissue and verifying the recovered DNA by qRT‐PCR. Our results show an apparent increase in the binding of Pparγ to the Obr CRE sites, suggesting that Pparγ promotes the transcription of Obr (Figure 8H).

## 3 Discussion

Herein, we report the lncRNA Obr, whose expression is developmentally regulated in the tissues of metabolic relevance, such as the skeletal muscle, liver, pancreas, and adipose tissue. Expression of Obr is also altered in diet‐induced obesity. Lipids, energy storage units, and signaling molecules are essential components of cells, tissues, and organisms. Of the different organs, two major organs that fulfil these functions are the liver and adipose tissues.^[^ ^34^ ^]^ Changes in lipid metabolism, the process by which lipids are synthesized for storage or broken down to release energy, are the primary cause of obesity. In obesity, an altered lipid metabolism results in increased accumulation of low‐density lipoprotein (LDL), cholesterol, very‐low‐density lipoprotein (VLDL), triglycerides, and a decrease in the level of high‐density lipoprotein (HDL).^[^ ^35^ ^]^

LncRNAs are a large and diverse group of regulatory RNAs that modulate gene expression through various mechanisms and contribute to different biological regulatory processes.^[^ ^36^, ^37^ ^]^ It has been postulated that each organism has thousands of lncRNAs. The RNA central database, which includes the lncRNAs from rats, shows 29693 lncRNAs for rats; out of these, Gene Ontology terms are known for only 292 of these lncRNAs, suggesting only a few lncRNAs which have been characterized so far (https://rnacentral.org). Although limited, these studies have shown the functional roles of lncRNAs in lipid metabolism by regulating the expression of key genes, networks, and pathways involving cholesterol and triglyceride biosynthesis, cholesterol transport, lipid uptake, and efflux.^[^ ^36^, ^38^ ^]^ It has been shown that the expression of the lncRNA HOXA1‐AS1 positively correlated with adipogenesis, and its expression significantly increased in obese patients. This is through its regulation of the adipogenic genes, including CEBP‐α, DGAT2, CIDEC, and perilipin.^[^ ^39^ ^]^ LncRNA AC092159.2 was shown to be a positive regulator of body mass index (BMI) and obesity through its regulation of TMEM18.^[^ ^40^ ^]^ Another inducer of lipid accumulation and insulin resistance, lncRNA MALAT1, was shown to function through stabilizing the protein complex, which included the critical regulator of lipid metabolism and fatty acid synthesis SREBP1c, which was increased in the liver of obese patients.^[^ ^41^ ^]^ In obese mice, a decrease in the expression of lncRNA U90926 was observed in the subcutaneous and visceral adipose tissue. Its overexpression in 3T3‐L1 reduced adipocyte differentiation through the negative regulation of PPARγ2.^[^ ^42^ ^]^ In obese mice and NAFLD patients, lncRNA Shgl and its human homolog lncRNA B4GALT1‐AS1 were shown to be down‐regulated.^[^ ^43^ ^]^ The expression of the lncRNA UC.372, which regulates the CD36‐dependent lipid uptake, was increased in the liver of db/db mice, high‐fat‐diet‐fed mice, and NAFLD patients.^[^ ^44^ ^]^ A role for lncRNA LeXis in regulating cholesterol biosynthesis through the liver X receptor (LXR) had also been reported.^[^ ^45^ ^]^ Our results show that Obr regulates genes involved in lipid metabolism by binding to the CREs as a part of the Creb complex.

CREB is a transcription factor that activates the transcriptional activity of different genes by binding to the CRE sequences and regulates adipogenesis in cells and tissues.^[^ ^13^, ^24^, ^27^, ^46^ ^]^ Our results show that the lncRNA Obr possesses multiple CRE‐like sequences, and Creb protein binds to Obr.

The different functions mediated by the CREB protein are orchestrated through the interactions of its domains with the interaction proteins.^[^ ^47^ ^]^ While the basic leucine zipper motif is essential for the dimerization and binding of the DNA, the kinase inducible domain has been suggested to be responsible for transcriptional activation. Through its kinase inducible domain, CREB is shown to interact with the histone acetyltransferases, CBP, and p300.^[^ ^14^, ^48^ ^]^ When phosphorylated at Ser133, CREB binds to the CREs, which are then bound by CBP and P300 acetyltransferases, which result in transcriptional activation, and this metabolic signaling process has been shown to promote adipogenesis.^[^ ^49^ ^]^ Our results also reveal that lncRNA *Obr* associates with the Creb histone acetyltransferase complex, Creb, Cbp, and p300, and positively regulates the transcription of genes involved in lipid metabolism as a part of the complex.

The histone tail modification histone 3 lysine 27 acetylation (H3K27ac), an indicator of transcriptionally active genes, is promoted by the histone acetyltransferases, CBP and p300.^[^ ^50^, ^51^ ^]^ It has also been suggested that the histone acetylation mediated through CBP/p300 improves the efficacy of transcription factor recruitment.^[^ ^52^ ^]^ The central acetyltransferase or the HAT domains of p300/CBP are shown to catalyze the enzymatic reaction where an acetyl group is transferred from acetyl‐CoA to the Lys ε‐amino residues of different proteins, including that of the histones.^[^ ^51^ ^]^ Our results show that the Obr associates with Creb, Cbp, and P300, and this association is with the acetyltransferase activity of these histone acetyltransferases, as seen with the increase in H3K27ac levels in the target gene promoters involved in lipid metabolism.

Consistent with the results of previous studies, we found an increase in lipogenic gene expression related to lipid accumulation. C/EBPs, including C/EBPα, β, and δ, are a basic leucine zipper family of transcription factors which are essential for the development of adipocytes and accumulation of lipids through their regulation of lipogenic enzyme, diacylglycerol acyltransferase 2 (DGAT2).^[^ ^53^, ^54^ ^]^ Further, the absence of either one or both C/EBPβ and C/EBPδ reduces the fat mass and adipogenesis in mice and cellular models.^[^ ^55^ ^]^

Studies have also shown that the ectopic expression of C/EBPβ induces PPARγ expression in NIH3T3 fibroblasts.^[^ ^56^ ^]^ C/EBPβ, by binding to the promoters of PPARγ and C/EBPα, has also been shown to induce other genes involved in adipogenesis.^[^ ^57^ ^]^ Studies have also shown that all these genes, C/EBP α, C/EBP β, and PPARγ, are targets of CREB.^[^ ^23^, ^56^ ^]^ Human gastrin‐releasing peptide receptor gene regulation requires transcription factor binding at two different CRE sites,^[^ ^20^ ^]^ and Obr regulates all these genes as part of the Creb histone acetyltransferase complex.

The transcription factor PPARγ is a master regulator of adipogenesis.^[^ ^58^ ^]^ In mice lacking PPARγ, adipogenesis is severely compromised with a lack of terminally differentiated adipose tissues, and they also developed fatty liver and lipodystrophy.^[^ ^59^ ^]^ Its ectopic expression activates the transcription of genes involved in adipogenesis.^[^ ^28^, ^54^, ^60^ ^]^ It has also been shown that the expression of PPARγ is increased in response to HFD in response to the presence of polyunsaturated fatty acids,^[^ ^61^ ^]^ and it mediates HFD‐induced lipid accumulation.^[^ ^31^ ^]^ It is also suggested that the polyunsaturated fatty acids in HFD induce Pparγ expression.^[^ ^32^ ^]^

Thus, the possible mechanism may be the increased lipids in the HFD or when there is increased lipid accumulation, there is an activation of Pparγ. Pparγ transcriptionally regulates the expression of Obr. Increased expression of Paprγ results in increased expression of Obr. Obr forms an increased Creb histone acetyltransferase complex. Once this is formed, it activates the genes involved in lipid metabolism, resulting in an increased accumulation of lipids (Figure 8I).

## 4 Experimental Section

### Cell Lines and Cell Culture Experiments

Clone 9 (C9) cells (ATCC CRL‐1439) rat liver epithelial cells were grown in DMEM/F12 media supplemented with 10% FBS, 1x penicillin/streptomycin. Cells were transfected with pRFP‐C‐RS‐Obr using the transfection reagent Viafect (Promega). Cells were selected with puromycin (4 µg mL^−1^) for 3 weeks, and RFP‐positive C9 clones were isolated for further functional characterizations. pRFP‐C‐RS vector control cells were also generated. All cell lines were maintained at 37 °C in a humidified, 5% CO2 atmosphere.

C9 cells were cultured in 6 well plates for 24 h before treating the cells with 10 and 100 µm GW9662 (MedChem Express), 100, and 150 µm Troglistazone (Sigma) or 50 and 100 µm arachidonic acid (Santa Cruz). The treatment was continued for 24 h, and the cells were harvested and analysed by quantitative RT PCR.

### Animal Experiments

Wistar rats were housed in individual cages in an air‐conditioned facility of the College of Medicine and Health Sciences, 22   °C ± 2  °C, with 55% humidity on a 12‐h light/dark cycle. All animals had free access to laboratory feed and pure water. At weaning, the male pups were separated into two groups, one feeding on normal chow and the other on a high‐fat diet (HFD; D12492, Rodent Diet with 60 kcal% fat). All experimental protocols for animal care, handling, and experimentation were approved by the Ethics Committee of the College of Medicine and Health Sciences of the UAE University (Nos ERA_2017_5634; ERA‐2019‐5979).

### Vectors and Antisense Oligonucleotides (ASOs)

1.387 Kb long lncObr cDNA was PCR amplified from RNA samples extracted from rat muscle using Phusion High‐Fidelity DNA Polymerase (M0530, NEB) and cloned at BamH1 site in p‐RFP‐C‐RS vector, and the sequence of the insert was verified by sequencing.

Antisense oligonucleotides (ASOs) were designed to target different regions of Obr RNA (Table S1, Supporting Information). ASOs were transfected at 10 pmoles mL^−1^ concentrations using Lipofectamine 3000 transfection reagent (L3000001, Thermofisher Scientific) in Opti MEM medium (31985062, Thermofisher Scientific). Cells were harvested 48 h post‐transfection for further analysis.

### RNA Isolation and Real‐Time PCR Analysis

Total RNA and real‐time RT‐PCR were performed as previously described.^[^ ^62^ ^]^ Briefly, total RNA was extracted using the Trizol method of RNA extraction, and 1 µg total RNA was converted to cDNA using ProtoScript II First Strand cDNA Synthesis Kit (E6560) in a reaction volume of 20 µL as per manufacturer's instructions (Applied Biosystems, USA). qPCR reactions were carried out using Luna Universal qPCR Master Mix (M3003L) with 200 ng of cDNA and 200 nm of each primer (listed in Table S2, Supporting Information) in a Quantstudio3 real‐time PCR system (Applied Biosystems, CA, USA). Relative gene expression was estimated using the GAPDH expression level as a reference.

### RT^2^ Profiler PCR Array

One µg total RNA was reverse transcribed and analyzed with Rat RT^2^ profiler PCR array for obesity (Qiagen, Cat#330231) following the manufacturer's instructions. Differentially expressed genes were further analyzed using pathfindR (an R package for comprehensive identification of enriched pathways)^[^ ^63^ ^]^ to identify the affected pathways. TRRUST v2 analysis^[^ ^64^ ^]^ identified the key transcription factors regulating the differentially expressed genes. Complex Heatmap (https://www.bioconductor.org/packages/release/bioc/html/ComplexHeatmap.html) was used to generate heatmaps, and scatter plots were drawn using ggplot2 (https://ggplot2.tidyverse.org/).

### Western Blot Analysis

Total protein was extracted using the RIPA lysis buffer (1x PBS, 50 mm NaF, 0.5% Na deoxycholate w/v, 0.1% SDS, 1% IGEPAL, 1.5 mm Na_3_VO_4,_ 1 mm PMSF and complete protease inhibitor; Roche Molecular Biochemicals, IN, USA). Cell and tissue samples were lyzed using the RIPA lysis buffer and centrifuged at 10 000 rpm for 10 min at 4 °C. The supernatant was collected and quantitated with Bio‐Rad protein micro assay using BSA as standard (Cat no. 500‐0001).

Western blot analysis was performed with 20 µg of total cellular protein extracts using antibodies against the following proteins CEBPα (8178 S, Cell Signalling Technology, CST), CEBPβ (3087S, CST), GRPR (ab39883, Abcam), PPARγ (Sc‐7273, Santa Cruz Biotechnology), p300 (ab275378, Abcam), CBP (ab235270, Abcam), CREB (9197S, CST), ‐CREB (phospho s‐133, ab32096, Abcam), and GAPDH (6C5) (sc‐32233; Santa Cruz). Secondary antibodies used were obtained from Bio‐Rad, USA.

### RNA EMSA

The RNA‐protein binding reactions were carried out using the LightShift Chemiluminescent RNA EMSA Kit (20158, ThermoFisher Scientific) following the manufacturer's protocol. Rat recombinant CREB protein for the binding assay was obtained from commercial sources (ab 43602, Abcam). Following the binding reactions, the RNA‐protein complexes were resolved on 6% native PAGE (ThermoFisher Scientific), transferred to N+ nylon membrane, and developed using the chemiluminescence detection module of the LightShift Chemiluminescent RNA EMSA Kit.

For RNA EMSA, biotinylated RNA probes:

Probe 1: UGAGAUUACCUGACGGCGAGUAAGCC,

Probe 2: CAGGUGUUCCUGACGGGGUCCUCAGA, Metabion International were used.

### RNA In Situ Hybridization

RNA in situ hybridization was carried out using FAM‐labeled RNA probes (lncRNA Obr probe, cat #339501: TAAATAGGCGATGTCCGCGCT, Control probe, Cat# 339508: GTGTAACACGTCATACGCCCA, Qiagen).

For this, C9 rat liver cells were fixed on slides and hybridized in the dark with the probes in 2× SSC in the presence of 60% deionized formamide, after which the slides were washed with 2× SSC.

For RNA in situ hybridization followed by immunohistochemistry after the hybridization, the cells were incubated with primary antibodies (CBP, MAB2676, R&D systems; p300, AF3789, R&D systems; and or pCREB, 9198s, Cell Signaling Technology) followed by secondary antibodies conjugated with fluorophores (RRX Donkey Anti‐Mouse IgG, 715‐295‐151; Cy5 Donkey Anti‐Goat IgG, 705‐175‐147; Cy5 Donkey Anti‐Rabbit IgG, 711‐175‐152; RRX Donkey Anti‐Goat IgG Jackson Immuno‐Research). Nuclei were stained with propidium iodide, and the slides were mounted using VECTASHIELD Antifade Mounting Medium) (H1300‐10, Vector Laboratories).

### Isolation of Nuclear and Cytoplasmic RNA Fractions

Nuclear and cytoplasmic RNA fractions were separated and extracted using the sub‐cellular RNA extraction kit (25501, Active Motif) following the manufacturer's protocol. Briefly, the cells were lyzed in a complete lysis buffer, incubated on ice for 10 min, and centrifuged to collect the supernatant (Cytoplasmic fraction). Both the supernatant and the pellet (containing nuclear fraction) were added with buffer G containing β‐Mercaptaethanol and extracted with 70% ethanol. RNA was purified from these extracts using the column method.

### RNA Immunoprecipitation

RNA immunoprecipitation assays were carried out following the protocol from Magna RIP RNA‐Binding Protein Immunoprecipitation Kit (Catalog no 17–700, Sigma–Aldrich). Briefly, 100 µL of cell lysate from 250 mg tissue or 15–20 million cells was incubated in RIP buffer with magnetic beads coated with anti‐CREB antibody or negative control IgG. The immunoprecipitated RNA was purified following Proteinase K digestion. RNA extracted from the beads was quantified by quantitative real‐time PCR analysis after normalizing to the input RNA quantities. Proteins were analyzed by western blot analysis. Proteins extracted from the inputs and immunoprecipitations using isotype antibodies were loaded as positive and negative controls, respectively.

### Chromatin Isolation Via RNA Purification (ChRIP)

ChRIP was carried out using the standard reagents and protocol from EZ‐Magna ChIRP RNA Interactome Kit‐Isolation and characterization of non‐coding RNA: chromatin complexes (17‐10495, Merck). Briefly, cells or finely chopped tissues were crosslinked with 1% glutaraldehyde in 1x PBS for 10 min. Cell lysates prepared from single‐cell suspensions were sonicated and hybridized with 300 picomoles of biotinylated DNA probes for 4 h at 37 °C, and the RNA‐protein–DNA complexes were isolated using streptavidin magnetic beads. Following washing, RNA, DNA, and proteins were separated from the complexes and analyzed by RT‐PCR or western blot analyses. 2% of the sonicated lysate was preserved before hybridization and used as input for analysis. A list of primers used for ChIRP analysis is given in Table S4, Supporting Information.

### Chromatin Immunoprecipitation Assay (ChIP)

ChIP was performed using the standard reagents and protocol from SimpleChIP Enzymatic Chromatin IP Kit (Magnetic Beads) (9003, Cell Signaling Technology). Briefly, cells or finely chopped tissues were crosslinked with 1.7% formaldehyde in 1× PBS for 20 min. Nuclei were isolated from cells and digested with micrococcal nuclease (MNase) at 37 °C for 25 min. Chromatin extracts separated from the digested nuclei by brief sonication, followed by centrifugation at 9400 × *g* for 10 min at 4 °C, were used for immunoprecipitation using the indicated antibodies at 4 °C overnight. 2% of the chromatin extracts were preserved as input for DNA analysis. Protein‐DNA complexes isolated using protein A/G magnetic beads were reverse crosslinked, and DNA was extracted. The DNA extracts were analyzed by RT‐PCR. For immunoprecipitation, acetyl‐Histone H3 (Lys27) antibody, acetylated lysine antibody, histone H3 antibody, Rabbit IgG (8173, 9441, 4620, and 2729, respectively from Cell Signaling Technologies), and pCREB antibody (PA1‐184, Invitrogen) were used. A list of primers used for ChIP analysis is given in Table S5, Supporting Information.

### Statistics

All data are expressed as means ± SEM of triplicate experiments. Data were analysed using the two‐tailed *t*‐test or ANOVA.

### Ethics Approval

All animal experiments were reviewed and approved by the Animal Research Ethics committee of UAE University, Nos ERA_2017_5634; ERA‐2019‐5979.

## Conflict of Interest

The authors declare no conflict of interest.

## Author Contributions

B.S.E. conceived the idea and designed the experiments. S.A.M., P.S., and S.A.A. contributed to the final design of the experiments. S.K., S.S.L., N.M., C.A.K., and P.S. performed the experiments. B.S.E. analyzed the results and wrote the paper with contributions from S.K., S.S.L., N.M., C.A.K., S.A.M., P.S., and S.A.A. B.S.E. secured the funds for this work. All authors reviewed, edited the manuscript, and approved the final manuscript.

## Supporting information

Supporting Information

## Data Availability

Data sharing is not applicable to this article as no new data were created or analyzed in this study.
