# Supplementary material for: The Long Non‐Coding RNA Obesity‐Related (Obr) Contributes To Lipid Metabolism Through Epigenetic Regulation
Source: Adv Sci (Weinh). 2024 May 5;11(26):2401939. doi: 10.1002/advs.202401939 (PMC11234455; doi:10.1002/advs.202401939)
Supplement: Supplementary file 1 — Supporting Information [file ADVS-11-2401939-s001.pdf]

ADVANCED SCIENCE
Open Access

Supporting Information

for *Adv. Sci.*, DOI 10.1002/advs.202401939

The Long Non-Coding RNA Obesity-Related (Obr) Contributes To Lipid Metabolism Through Epigenetic Regulation

*Suneesh Kaimala, Shareena Saeed Lootah, Neha Mehra, Challagandla Anil Kumar, Saeeda Al Marzooqi, Prabha Sampath, Suraiya Anjum Ansari and Bright Starling Emerald**

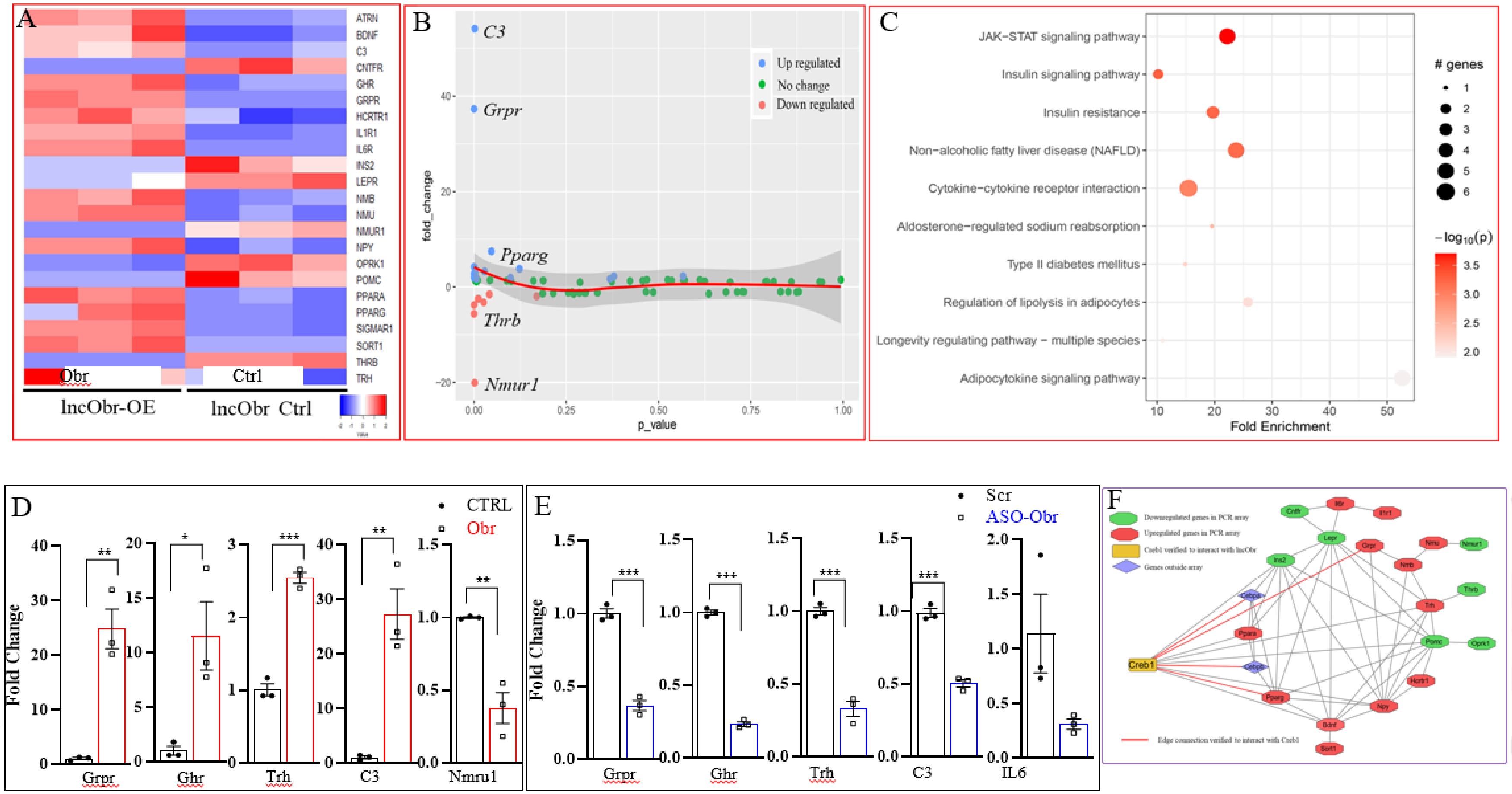

Figure S1. Identification and verification of genes involved in lipid metabolism which are altered by lncRNA *Obr* using RT2 Profiler rat obesity PCR array

A. Heat maps of PCR array analyses of C9 cells that stably overexpress *Obr* reveal that Obr alters lipid metabolism genes. The expression of each gene was normalised to the expression of the housekeeping genes B2M, GAPDH and HSP90AB1. Data are presented as fold change and a $P<0.05$ cut-off for statistically significant DEGs. Genes with higher expression levels are depicted in red, whereas genes with lower levels are shown in blue. B. Scatter plot of RT2 Profiler rat obesity PCR array results with up/down-regulated genes, p-value < 0.05. C. Go terms, the number of genes, p-values and the pathways that are altered by overexpression of *Obr*. D-E. Verification of the genes of PCR array with changes in their expression by qRT-PCR analysis. Results of C9 cells with overexpression of (D) and downregulation of *Obr* (E) showing the expression changes. All qRT-PCR experiments were performed three times (n=3) in triplicate. F. The association of those genes changed in response to changes in the expression of *Obr*. Data are mean ± SE. $^{*}p<0.05$, $^{**}p < 0.01$, $^{***}p < 0.001$.

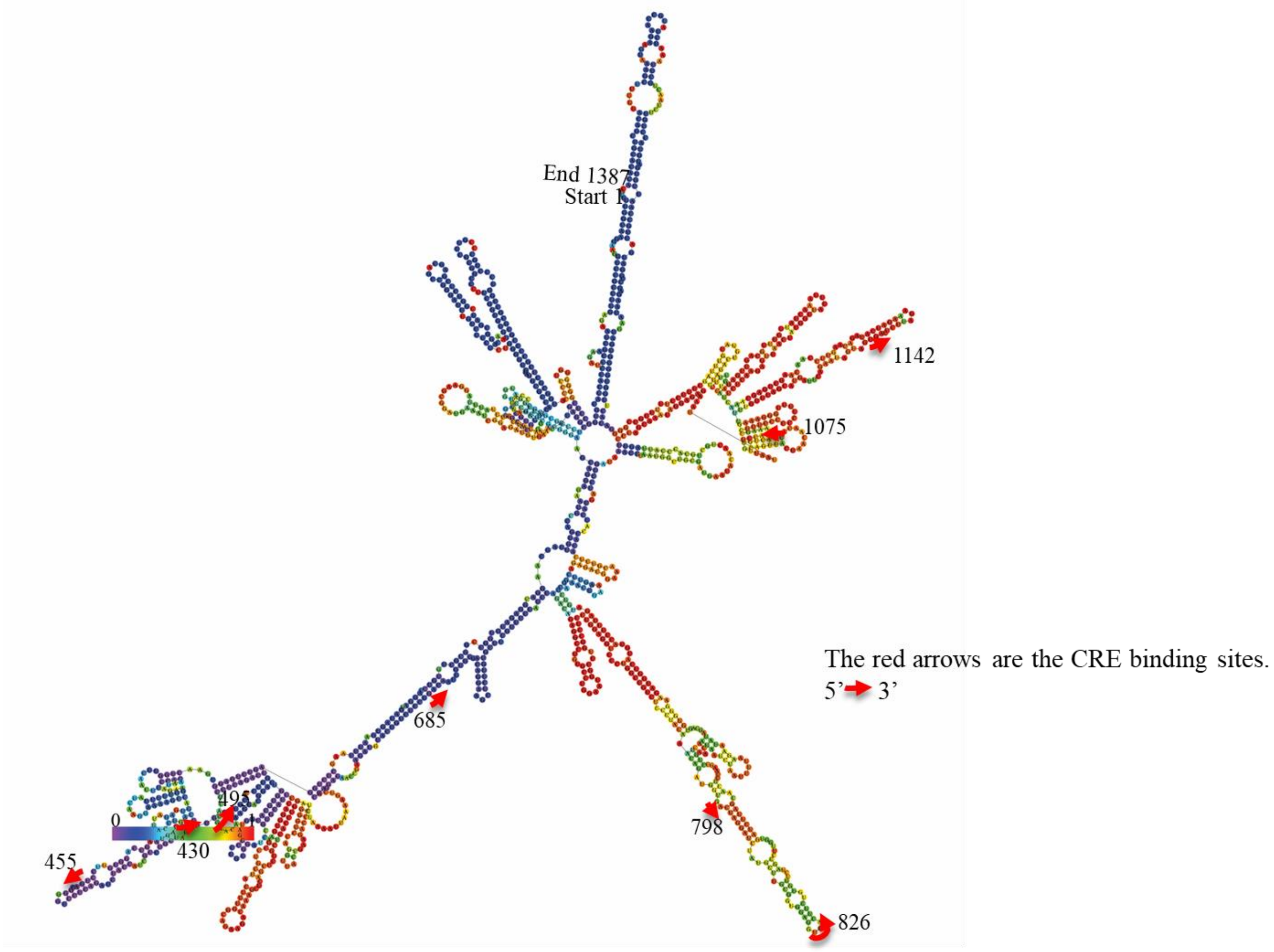

Figure S2

Obr secondary structure with the identified half CRE binding sites

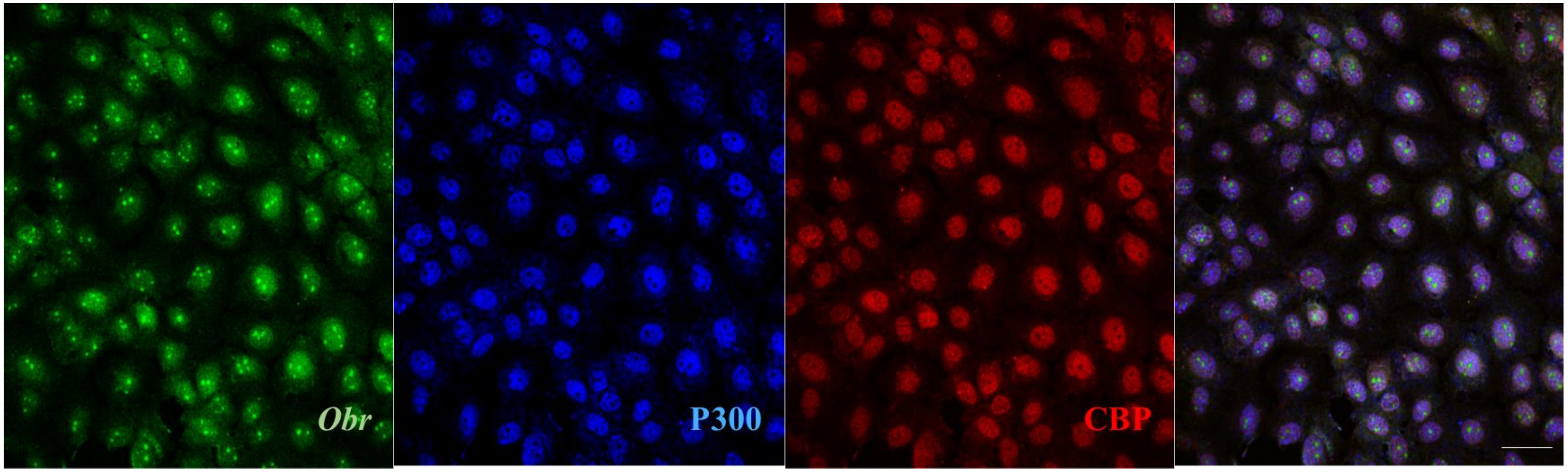

Figure S3

Co -localisation of the lncRNA *Obr* with that of the histone acetyltransferases p300 and Cbp.

C9 cells we probed for the expression of Obr using in situ hybridisation followed by immuno staining for the histone acetyltransferases, p300 and Cbp using specific antibodies. *Obr* was colocalized with p300 and Cbp in the nucleus. Scale bar = 50 μm.

Sequence name	Sequence	DNA/2-O=methyl RNA Chimera
709-ASO1	GCGATTCTCAAGGCGGACC	mG*mC*mG*mA*mU*T*C*T*C*A*A*G*G* C*mG*mG*mA*mC*mC*
709-ASO2	AGGTGAGCAGAGCTGAGCT	mA*mG*mG*mU*G*A*G*C*A*G*A*G*C*T *mG*mA*mG*mC*mU*

Table S1

The list of Antisense oligonucleotides (ASOs) used to target different regions of *Obr* RNA

Sl No	Name	Sequence
1	Rn_110709 full	F: CGTTTTGGTAAGGAAGGTCCGCCTTGAGAATCGC<br>R: TGTGTGCAGCTGTAAAAAGCTTTACTTCTTGGC
2	Rn_Pparg	F: GGACGCTGAAGAAGAGACCTG<br>R: CCGGGTCCTGTCTGAGTATG
3	Rn_Trh	F: ATTGCAAACTCTACCCAGCCA<br>R: TGGAGTCTGCGAAGTGGAGA
4	Rn_Grpr	F: GTGAACCCCTTTGCCCTGTA<br>R: ATGCAGGTGGTACTTCTGCC
5	Rn_ Ghr	F: TGCTACAGACCAAGACACCAAG<br>R: AGAACACCCGCCAAAGATCC
6	Rn_ C3	F: ATCGAGGATGGTTCAGGGGA<br>R: GCCTCTACCATGTCGCTACC
7	Rn_NmuR1	F: GACTTCCCCTGCAGAAGGCAT<br>R: TCCTCAAGGTCAAAGTGCTCC
8	Rn_IL6R	F: CCCCATCAGGGTCCCATAAC<br>R: AGTGAGAAACTTGCGTGCCA
9	Rn_GAPDH	F: TGTGAACGGATTTGGCCGTA<br>R: GATGGTGATGGGTTTCCCGT

Table S2

The list of qRT-PCR primers used in the study

ChRIP probe	Sequence
Obr ChIRP probe_1	CAGCTCTTATTCATCACCCCACCAAGTGTCCCCGGCAAAAAGGAAAGCTA
Obr ChIRP probe_2	CTGTCGCACAATTGTCAGGAGACAGTACATCTTCGGTTCTCCTGGTGACT
Obr ChIRP probe_3	CTGTCGCACAATTGTCAGGAGACAGTACATCTTCGGTTCTCCTGGTGACT
Rn_Gapdh Probe 1	GAGGGTGCAGCGAACTTTATTGATGGTATTCGAGAGAAGGGAGGGCTCCC
Rn_Gapdh Probe 2	AGAGCAATGCCAGCCCCAGCATCAAAGGTGGAAGAATGGGAGTTGCTGTT
Rn_Gapdh Probe 3	TCTGGGCTGCCCCACGGCCATCACGCCACAGCTTTCCAGAGGGGCCATCC
Rn_Gapdh Probe 4	CATGGTGGTGAAGACGCCAGTAGACTCCACGACATACTCAGCACCAGCAT

Table S3

List of probes used for the ChRIP analysis

ChRIP Primers	Sequence	Region covered	Length of PCR product (bp)
Rn_ CEBPa CREB -1108 F	TTCAGGCCCCTGACTATGGG	-991 to -1108 bp upstream of C/ebp α gene	117
Rn_ CEBPa CREB -991 R	ACAAGACACTAAGGAGACCGC		
Rn_ CEBPb CREB -4860 F	ATGGCGTAGGCACCAATCAT	-4730 to -4860 bp upstream of C/ebp β gene	130
Rn_ CEBPb CREB -4730 R	ACATCTTCCTGTGCCAGCAT		
Rn_ GRPR CREB -651 F	TAGGAGTGGCGATCTGCTCC	-578 to -651 bp upstream of Grpr gene	73
Rn_ GRPR CREB -578 R	GCTGGACGCTTCAATCACTG		
Rn_ Pparg CREB -509F	AGCACCAACCAACCACTTGT	-367 to -509 bp upstream of Ppar γ gene	142
Rn_ Pparg CREB -367 R	GAAGGAGAACACCTTCATAAACTGA		
Rn_ Pparg CREB -1308F	CGGAACTCCTGCATGAGCAAG	-1232 to -1308 bp upstream of Ppar γ gene	76
Rn_ Pparg CREB -1232 R	CTCAGCGGGAAGTACGCATGT		

Table S4

List of primers used for ChIRP analysis

ChIP Primers	Sequence	Region covered	Length of PCR product (bp)
Rn CEBPα Ac -2370 F	CCATTTCATGGGCGATGGCT	-2234 to -2370 bp upstream of C/ebp, α gene	136
Rn CEBPα Ac -2234 R	GGGGAGCATGAGAAGTGTCC		
Rn CEBPβ Ac -665 F	AGACACAGTGTGGCAGAACC	-567 to -665 bp upstream of C/ebp β gene	98
Rn CEBPβ Ac -567 R	TCAACCCTTTGTATGGCCCG		
Rn GRPR Ac -399 F	ACGGGGAAATGCTGTGTGAG	-578 to -651 bp upstream of Grpr gene	73
Rn GRPR Ac -231 R	ACACCACCAGCAGGCAATAC		
Rn PPARγ Ac -15006 F	CCCCCACGTTGTCAGACATT	-14903 to -15006 bp upstream of Ppar γ gene	103
Rn PPARγ Ac -14903 R	GGAGGTGTTCCTCACAGGTT		
Rn PPARγ Ac -215 F	CCATGGGCATCTGTCTGAGG	-164 to -215 bp upstream of Ppar γ gene	51
Rn PPARγ Ac -164 R	CGCCTTGCTCCTCACAGTC		
Rn PPARγ Ac -736 F	AGGCTCCTGAGACAAGATGC	-645 to -736 bp upstream of Ppar γ gene	91
Rn PPARγ Ac -645 R	CTTGCATGGGGGACCTTACA		

Table S5

List of primers used for ChIP analysis
